# 1,25-Dihydroxyvitamin-D_3_ Induces Avian β-Defensin Gene Expression in Chickens

**DOI:** 10.1371/journal.pone.0154546

**Published:** 2016-05-02

**Authors:** Long Zhang, Lu Lu, Siming Li, Guolong Zhang, Linghua Ouyang, Kelsy Robinson, Yanqiang Tang, Qing Zhu, Diyan Li, Yaodong Hu, Yiping Liu

**Affiliations:** 1 Farm Animal Genetic Resources Exploration and Innovation Key Laboratory of Sichuan Province, Sichuan Agricultural University, Chengdu, 611130, P. R. China; 2 Institute of Animal Husbandry and Veterinary, Jiangxi Academy of Agricultural Science, Nanchang, 330200, P. R. China; 3 Department of Animal Science, Oklahoma State University, Stillwater, Oklahoma, 74078, United States of America; Kunming Institute of Zoology, Chinese Academy of Sciences, CHINA

## Abstract

Host defense peptides (HDPs) play a critical role in innate immunity. Specific modulation of endogenous HDP synthesis by dietary compounds has been regarded as a novel approach to boost immunity and disease resistance in animal production. 1,25-dihydroxy vitamin D_3_ (1,25D_3_) is well known as a powerful HDP inducer in humans, but limited information about the effect of 1,25D_3_ on HDPs in poultry is available. Here, we sought to examine whether 1,25D_3_ could stimulate avian β-defensin (AvBD) expression in chickens. We used chicken embryo intestinal epithelial cells (CEIEPCs) and peripheral blood mononuclear cells (PBMCs) to study the effect of 1,25D_3_ on the expression of AvBDs. We observed that 1,25D_3_ is able to up-regulate the expression of several AvBDs in CEIEPCs and PBMCs, whereas it increased the amounts of *AvBD4* mRNA in CEIEPCs only in the presence of lipopolysaccharide (LPS). On the other hand, LPS treatment not only inhibited the expression of *CYP24A1* but also altered the expression pattern of *VDR* in CEIEPCs. Furthermore, AvBDs were not directly regulated by 1,25D_3_, as cycloheximide completely blocked 1,25D_3_-induced expression of AvBDs. Our observations suggest that 1,25D_3_ is capable of inducing *AvBD* gene expression and is a potential antibiotic alternative through augmentation of host innate immunity as well as disease control in chickens.

## Introduction

Host defense peptides (HDPs) are a group of cationic amphipathic peptides with less than 100 amino acid residues. They are important immune molecules that are able to kill a broad range of microbes, including viruses, Gram negative and Gram positive bacteria, fungi, protozoa and parasites. Besides those broad-spectrum antibiotic activities, they also play important roles in adaptive immunity, wound healing and sperm fertilization [1–3]. In birds, avian β-defensins (AvBDs) are the biggest cluster of host defense peptides, these peptides consist of a signal peptide, short propiece and conserved mature peptide. There are a total of 14 chicken AvBDs located on chromosome 3 and expressed in a wide array of tissues [4–6].

In order to meet the increasing demands for meat, antibiotics have been used in animal production since 1950s. Although antibiotic use is an effective way of improving animal feed efficiency, preventing disease, and controlling infections, routine use of antibiotics in-feed has been criticized for triggering the emergence of drug-resistant microbes and contamination of food products and environment with unwanted antibiotic residues [7–9]. Numerous countries have implemented bans on the use of low dose of antibiotics as feed additive [10]. Therefore, antibiotic alternatives are urgently needed to ensure animal health and food safety. HDPs are attractive candidates as alternatives to antibiotics as they have the potential to control a broad spectrum of pathogens without eliciting resistance. However, high production cost and peptide instability limit direct use of HDPs as effective antibiotics alternatives in animals [1]. Recently, several nutrients have been found to be highly potent in augmenting HDP synthesis and enhancing disease resistance [11–15], suggesting a high-efficiency and low-cost strategy for antimicrobial therapy. Furthermore, because those compounds come from dietary source and have no direct interaction with pathogens they provoke no inflammatory response. Hence, dietary supplementation of HDP-inducing compounds is considered as a high efficiency strategy to enhance animal immunity.

Vitamin D_3_ (Vit-D_3_), also called cholecalciferol, is a sterol that can be obtained from dietary sources. After absorbed, the bioactive mechanism of dietary Vit-D_3_ contains two hydroxylated steps. The initial hydroxylation (25-hydroxylase) takes place in liver, where 25-hydroxycholecalciferol (25OHD_3_) is formed. The final hydroxylation occurs in kidney, bone, prostate and immunocytes by 1α-hydroxylases which convert 25OHD_3_ into 1,25-Dihydroxyvitamin-D_3_ (1,25D_3_), the most biologically active hormonal metabolite of Vit-D_3_ [16, 17]. Apart from its typical action in regulating calcium phosphorus homeostasis and bone mineralization, 1,25D_3_ is known to enhance the expression of HDPs and strengthen host innate immunity in various species. In human, 1,25D_3_ dramatically induce the expression of *cathelicidin* (LL-37) and defensins in different cell types [11, 18]. In bovine, although no evidence shows that 1,25D_3_ stimulate the production of cathelicidins, the expression of several HDPs such as β-defensins [19, 20] *S100A7* [20] and *S100A12* [21] are increased by 1,25D_3_. In chickens, previous studies showed that feed or injection of VIT-D_3_ can induce the expression of some HDPs in different tissues and conditions *in vivo* [22–24]. However, there is no direct evidence to indicate that Vit-D_3_ boost the expression of AvBDs through 1,25D_3_. Likewise, to our knowledge, there is no report about the effect of 1,25D_3_ on the expression of chicken AvBDs *in vitro*. In the present study, we report 1,25D_3_ is capable of inducing the expression of AvBDs in chicken embryo intestinal epithelial cells (CEIPCs) and peripheral blood mononuclear cells (PBMCs). Furthermore, we reveal that 1,25D_3_-induced expression of AvBDs in chickens is a secondary response to 1,25D_3_. These findings are vital in the development of antibiotic alternatives and disease prevention in poultry production.

## Materials and Methods

### Cell culture and treatments

CEIEPCs were prepared and cultured according to a previous method [25]. Briefly, 15-days-old chicken embryos were obtained from Beijing Merial Vital Laboratory Animal Technology Co, LTD. Beijing, China. Small intestines were separated and cut into small pieces prior to being digested with type I collagenase (200 U/mL, Sigma Aldrich) and hyaluronidase (100 U/mL, Sigma Aldrich) at 37°C. Then the cells were collected by centrifugation at 500×g for 10 min. After that, all cells were resuspended in 10 mm plates and incubated in complete DMEM/F12 (GIBCO) supplemented with 10% fetal bovine serum (Invitrogen), 20 μg/L epidermal growth factor (Sigma Aldrich), 100 mg/L heparin sodium (Sigma Aldrich), 110 mg/L pyruvate (Sigma Aldrich), 2.5 mg/L insulin (Sigma Aldrich), 200 mM glutamic acid (Sigma Aldrich), 100 U/L penicillin (Hyclone) and 100 μg/L streptomycin (Hyclone) for 24h. Last, cells were seeded in 6-well plates at a density of 5×10^5^ and cultured in 37°C under 5% CO_2_ overnight, prior to stimulation. PBMCs were isolated and cultured as previous descripted [12]. For signaling studies, PBMCs were pretreated with 10 μg /mL of cycloheximide (CHX) for 1h, followed by stimulation with 1.25D_3_. All procedures were approved by the Institutional Animal Care and Use Committee of the Sichuan Agricultural University under permit number DKY-S20123120. Lipopolysaccharide (LPS) from Escherichia coli 055:B5 (Sigma Aldrich) was solubilized in endotoxin-free water. 1,25D_3_ (Sigma Aldrich) was dissolved and diluted to different concentrations by 100% ethanol. For each treatment, 1 μL of solution with different doses of 1,25D_3_ was added into the cell culture medium. Cycloheximide (Santa Cruz) was also diluted in 100% ethanol and equal amount of 100% ethanol alone was added to the controls to balance the effect of ethanol.

### CCK-8 viability assay

Cell viability was assessed by using CCK-8 (Shanghai Bestbio Biotechnology Co. Ltd, Shanghai, China). CEIEPCs were seeded at a density of 5×10^4^ cells/well in 96-well plates. At the indicated times, CCK-8 was added and incubated for 2h at 37°C for color development. The degree of the color was directly proportional to the number of viable cells. The absorbance at 450 nm was measured using a microplate reader.

### RNA extraction and real-time PCR

Total RNA was isolated by RNAiso Plus (Takara Bio Inc., Dalian, China) and dissolved in RNase-free water. PrimeScript® RT reagent Kit with gDNA Eraser (Takara Bio Inc., Dalian, China) was used for reverse transcription reaction and performed according to the manufacturer’s instructions. Primer sequences, annealing temperature and accession numbers are shown in Table 1. The total volume of PCR contained 1 μL of cDNA, 0.8 μL 10 pmol/μL of each primer, 12.5 μL SYBR® Premix Ex Taq™ II (Takara Bio Inc., Dalian, China) and double-distilled H_2_O to 25 μL. The optimum thermal cycling procedure was 95°C for 2min, 40 cycles of 95°C for 5s, n°C for 30s (n is the annealing temperature), and 60°C for 30s. *GAPDH* was used as the reference gene and relative quantification of mRNA transcripts was accomplished using the 2^-ΔΔCt^ method [26]. For each experiment, the control samples were used as the calibrator, and expression of each gene is reported as fold increase relative to the controls.

**Table 1:** Detail information of primers used in real-time PCR analysis.

Primer	Forward primer	Reverse primer	Fragment length (bp)	Annealing Temperature (°C)	Accession numbers
*AvBD-1*	GGATGCACGCTGTTCTTGGT	TCCGCATGGTTTACGTCTGTC	100	60	NM_204993.1
*AvBD-2*	CTGCTTCGGGTTCCGTTCCT	TGCTGCTGAGGCTTTGCTGTA	127	60	DQ677633.1
*AvBD-3*	AGGATTCTGTCGTGTTGGGAGC	TTCCAGGAGCGAGAAGCCAC	143	62	NM_204650.2
*AvBD-4*	GGCTATGCCGTCCCAAGTATT	CCAAATCCAACAATGCAAGAAG	106	60	NM_001001610.2
*AvBD-5*	AGCCGATGGTATTCCTGATGG	TGGTGATTGTTGCCTCTGGTG	107	61	NM_001001608.2
*AvBD-6*	TGGCAGTGGACTAAAATCTTGC	TTTCACAGGTGCTGATAGGGA	197	59	NM_001001193.1
*AvBD-7*	ATGGAATAGGCTCTTGCTGTG	GCCAGATAGAATGGAGTTGGAG	119	58	NM_001001194.1
*AvBD-8*	CGACTAATGTTCGCCAGGACC	TCTCTTCTGTTCAGCCTTTGGTG	154	61	NM_001001781.1
*AvBD-9*	AACACCGTCAGGCATCTTCACA	CGTCTTCTTGGCTGTAAGCTGGA	131	62	NM_001001611.2
*AvBD-10*	AACTGCTGTGCCAAGATTCCG	AGGAGGAATCCATCACAATCAGC	112	62	NM_001001609.1
*AvBD-11*	AGTCTGCAATTCGTTAGAGGCG	GGATGTGGTTTCCAAGGGTTTA	180	61	NM_001001779.1
*AvBD-12*	CACCAACTCCCACCAAGACCT	AAGTGAATCCACAGCCAATGAGA	144	60	NM_001001607.2
*AvBD-13*	AGCTGTGCAGGAACAACCATG	CAGCACTGAATGTTTAGGGTTGG	143	60	NM_001001780.1
*AvBD-14*	TGTCGGAAGATGAAGGGCAA	GCCAGTCCATTGTAGCAGGT	83	59	AM402954.1
*CYP24A1*	CGGTAGAAACGCTGCATTCAG	TAGGGCCGTCATTAGTCAAGC	94	60	NM_204979.1
*VDR*	AGAAGCAAATTCAGCAGCAGGA	AAGGCATCGGAGCCAAAGAC	101	60	NM_205098.1
*IL-1β*	ACTGGGCATCAAGGGCTA	GGTAGAAGATGAAGCGGGTC	131	56	NM_204524.1
*TLR-4*	ACTGTCAAGGCTGAGAACGG	AGCTGAGGGAGCTGAGATGA	204	58	NM_204305.1
*GAPDH*	CCAGAACATCCCAAGCGTC	GGCAGGTCAGGTCAACAACAGA	134	60	NM_204305.1

### Data Analysis

All data is shown as Means ± standard deviation (SD). Statistical analyses were performed by using SAS (version9.3) software and differences in ΔΔCT values among the different treatments were analyzed by one-way ANOVA, followed by the Tukey tested. The *P* values < 0.05 were considered statistically significant.

## Results

### Expression of target genes in CEIPCs

**Fig 1:**
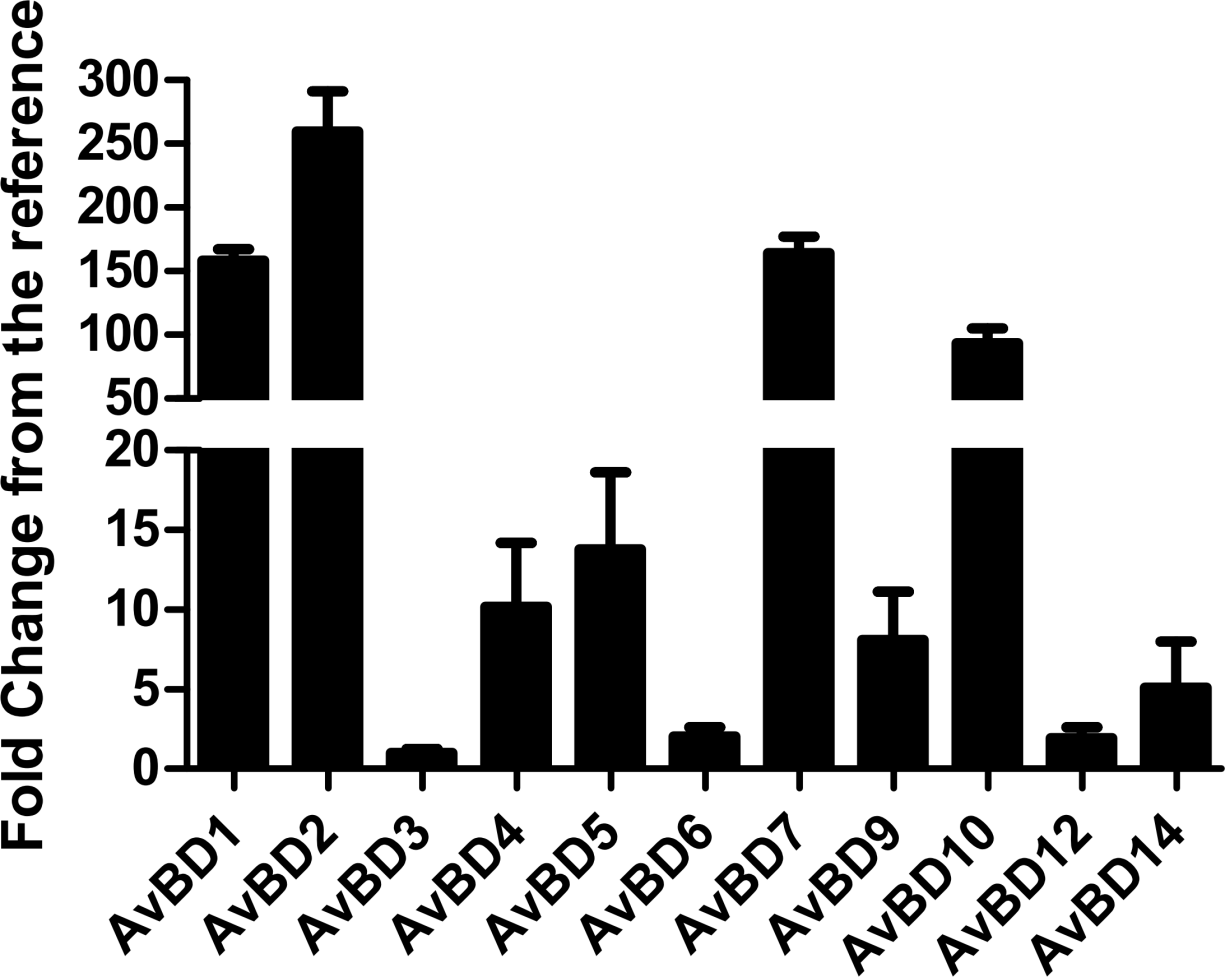
Relative expression levels of AvBDs in CEIEPCs. CEIEPCs were incubated with complete medium for 12h, followed by RNA isolation and real-time RT-PCR analysis of all 14 AvBDs. Expression levels of all AvBDs were calculated relative to that of *AvBD3* using GAPDH as a reference gene. Each bar represents mean ± SD of the results from two independent experiments performed in triplicate. *AvBD8*, *AvBD11*, and *AvBD13* were not reliably detected within 40 real-time PCR cycles, and therefore, were not shown.

**Fig 2:**
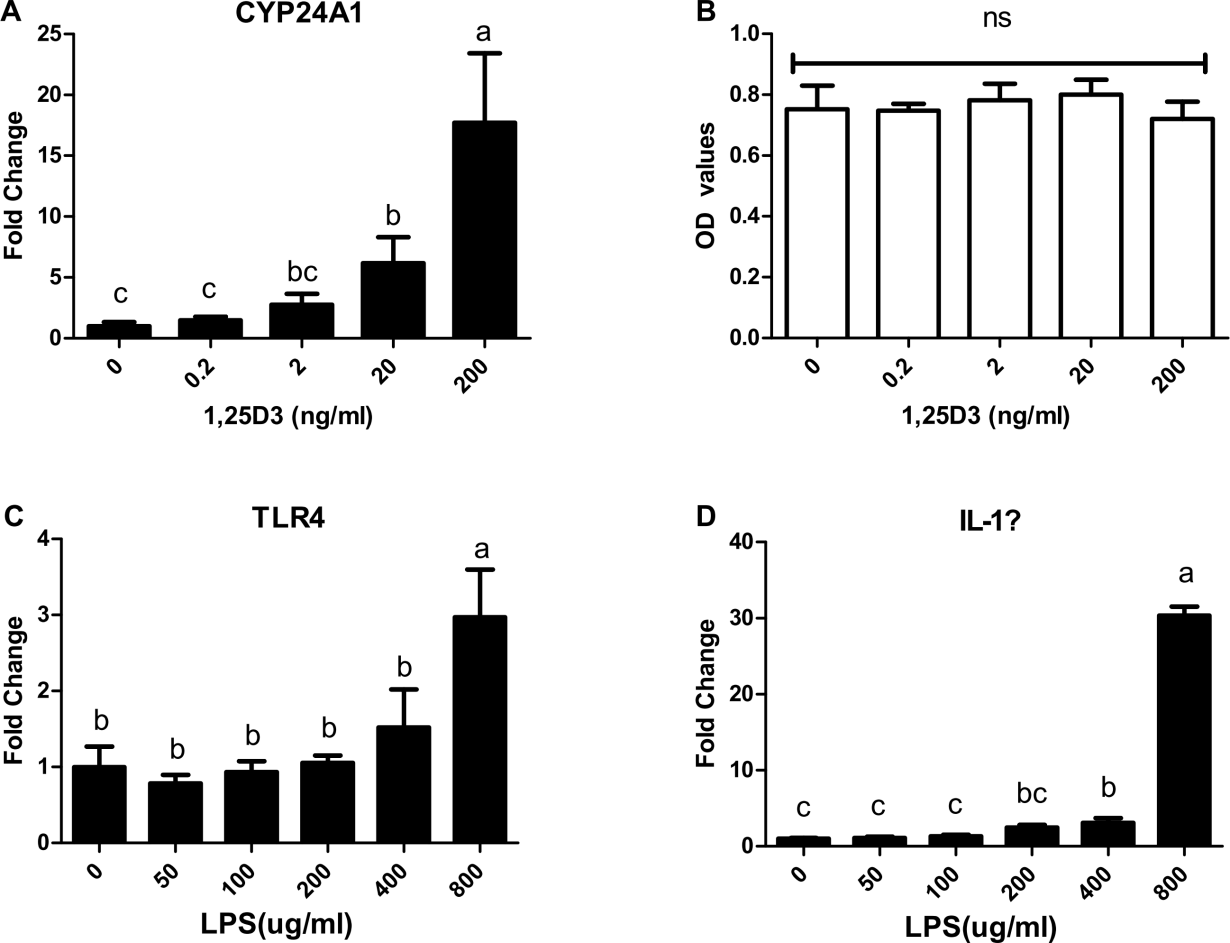
Effect of 1,25D_3_ on the expression of *CYP24A1* (A) and the growth (B) of CEIEPCs. CEIEPCs were incubated with indicated concentrations of 1,25D_3_ for 12h. Effect of LPS on the expression of *TLR4* (C) and *IL-1β* (D) in CEIEPCs. CEIEPCs were incubated with indicated concentrations of LPS for 12h. Data are shown as mean ± SD from 2–3 independent experiments. The bars without the same letter indicate differences significant at *P* < 0.05.

Prior studies in our lab demonstrated that injection of Vit-D_3_ is able to modulate AvBD gene expression in intestinal tissues [23]. To confirm the previous results, CEIEPCs were used as a model to evaluate the action of 1,25D_3_ on the expression of AvBDs with or without LPS. Basal expression levels of AvBDs are shown in (Fig 1). The expression of *AvBD8*, *AvBD11* and *AvBD13* was not detected in CEIEPCs within the 40 real-time PCR cycles. By contrast, the other 11 AvBDs were expressed in chicken CEIEPCs (Fig 1). Of those, the mRNA of *AvBD1*, *AvBD2* and *AvBD10* were more abundant, while *AvBD3*, *AvBD6* and *AvBD12* were expressed at lower levels. In order to ensure CEIEPCs are responsive to 1,25D_3_ and LPS, cells were treated with increasing doses of 1,25D_3_ or LPS. As illustrated in Fig 2A, 1,25D_3_ influenced 24-hydroxylase (*CYP24A1*) expression in a dose-dependent manner. Compared with controls, 200 ng/mL of 1,25D_3_ robustly induced *CYP24A1* (>15 fold) expression in CEIEPCs, while the cell viability was not significantly influenced by different concentrations of 1,25D_3_ (*P* > 0.05) (Fig 2B). The expression of *toll-like receptors* (*TLR*)*-4* and *interleukin* (*IL*)-*1β* were not significantly up-regulated by LPS until its concentration reached to 800 μg /mL and 400 μg/mL, respectively (Fig 2C and 2D). Since 800 μg/mL is a relative high dose of LPS concentration, we did not try a higher dose of LPS in this study.

### Effect of 1,25D_3_ combined with LPS on the expression of AvBDs in CEIEPCs

**Fig 3:**
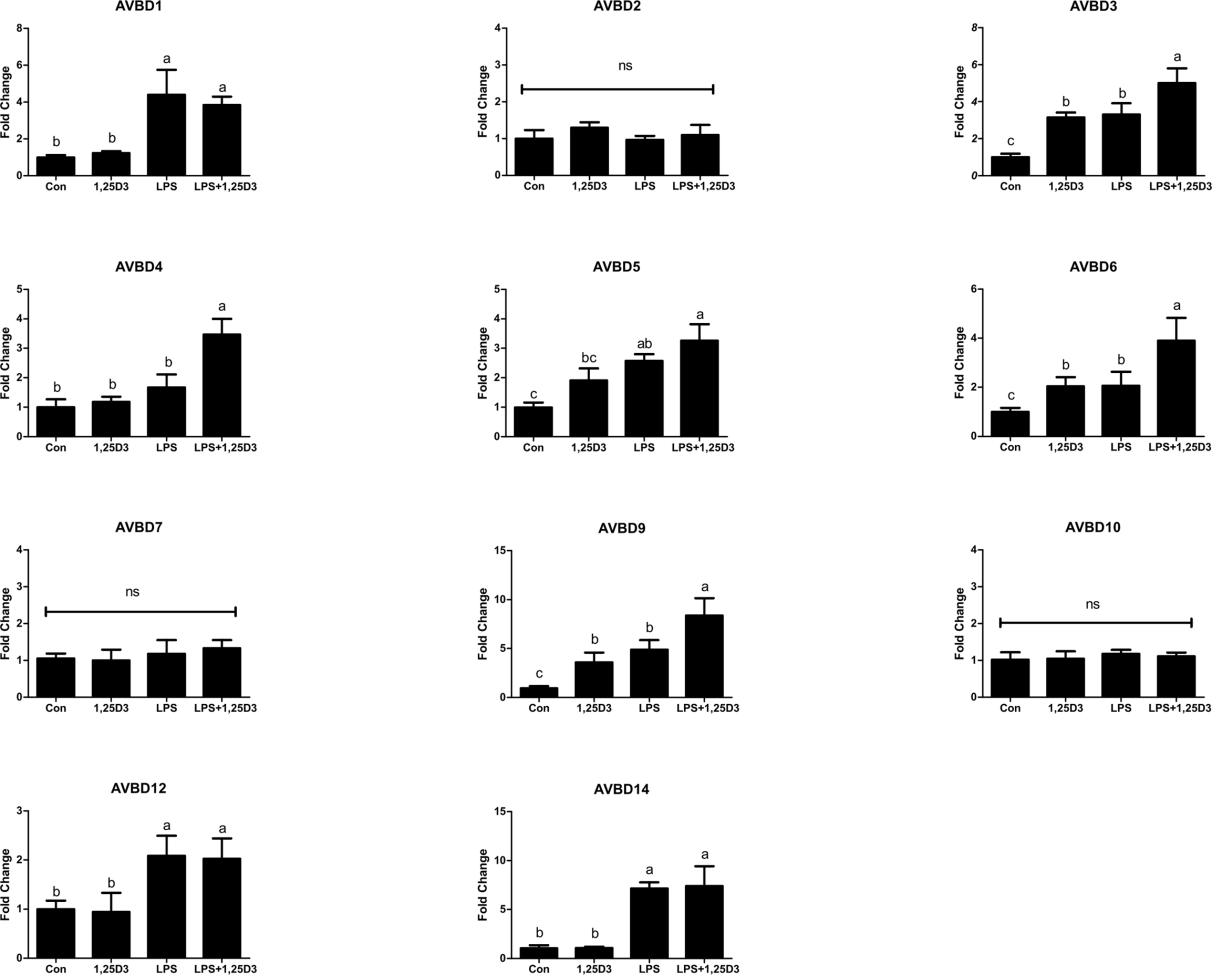
Effects of 1,25D_3_ on the expression of AvBDs with or without the presence of LPS. CEIEPCs were treated with 20 ng/mL of 1,25D and 800 μg/mL for 12h. The relative gene expression was measured by qPCR and normalized to *GAPDH*. Each bar represents mean ± SD of the results from 2–3 independent experiments performed in triplicate. The bars without the same letter indicate differences significant at *P* < 0.05.

Along with the above analysis, CEIEPCs were treated with 20 ng/mL of 1,25D_3_ alone or in conjunction with 800 μg/mL LPS for 12h, and the expression of AvBD mRNA was measured by real-time PCR. The relative mRNA abundance of 11 AvBDs is summarized in Fig 3. Similar to the observations in other species, 1,25D_3_ induced the expression of several AvBDs with or without LPS. More specifically, the expression of *AvBD3*, *AvBD6* and *AvBD9* was significantly induced by 1,25D_3_ both in non-stimulated and LPS-stimulated CEIEPCs. Interestingly, the expression of *AvBD4* was not up-regulated by 1,25D_3_ treatment alone, while the combination of LPS and 1,25D_3_ increased *AvBD4* gene expression relative to either treatment alone.

**Fig 4:**
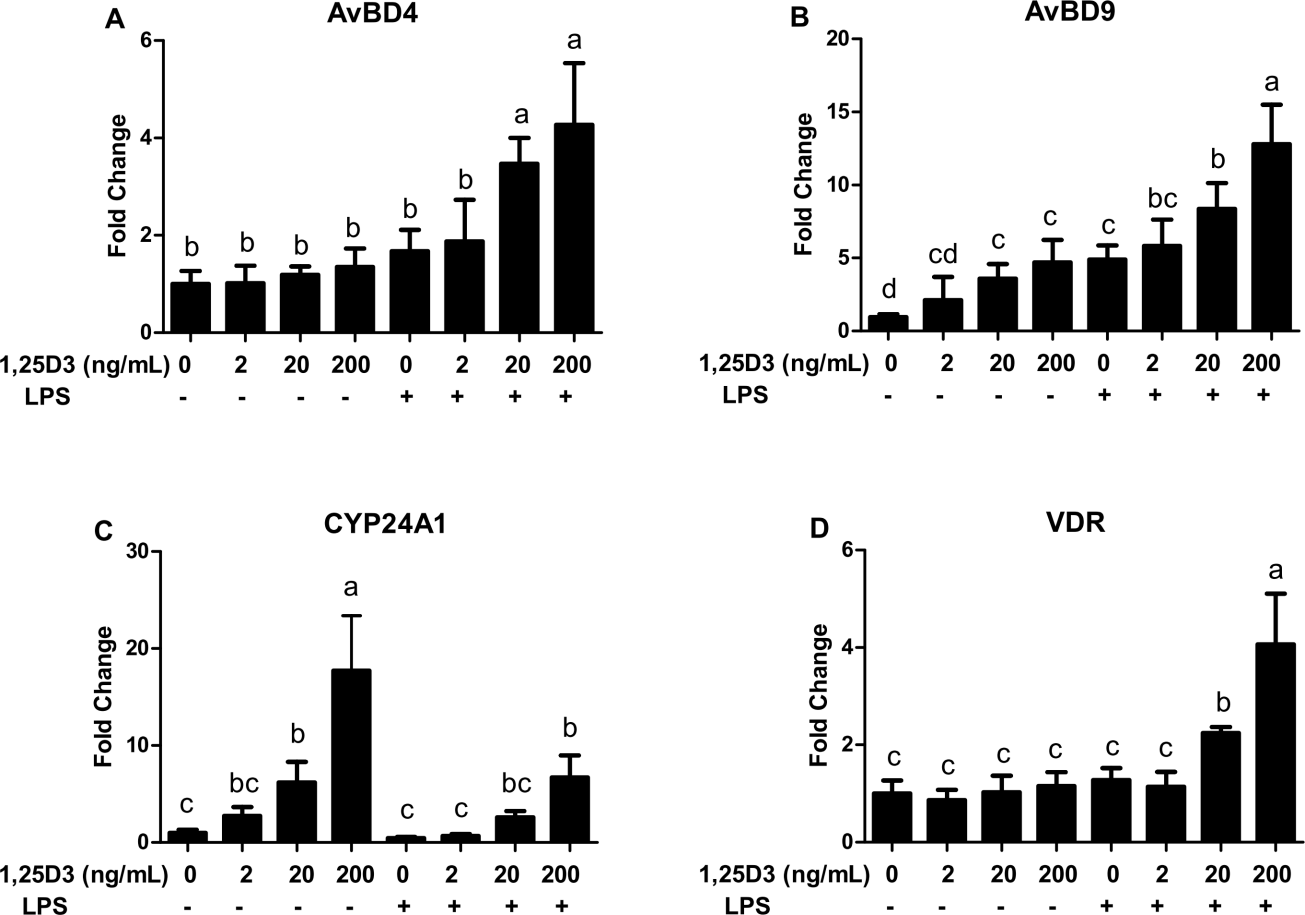
The synergy effect of 1,25D_3_ and LPS on the expression of *AvBD4* (A), *AvBD9* (B), *CYP24A1* (C) and the *VDR* (D). CEIEPCs were incubated with indicated concentrations of 1,25D_3_ with or without 800 μg/mL LPS for 12h. Data are shown mean ± SD from 2–3 independent experiments. The bars without the same letter indicate differences significant at *P* < 0.05.

To further investigate the HDP-inducing activity of 1,25D_3_, CEIEPCs were incubated with increasing concentrations of 1,25D_3_ with or without LPS stimulation. Expression of *AvBD4*, *AvBD9*, vitamin D receptor (*VDR*) and the positive control gene *CYP24A1* mRNA was evaluated by qPCR. Confirming our observations, the expression of *AvBD4* was up-regulated by 1,25D_3_ only in the presence of LPS (Fig 4A). *AvBD9* showed a dose-dependent expression in response to both 1,25D_3_ alone and synergy with LPS (Fig 4B). Moreover, LPS treatment significantly antagonized the expression of *CYP24A1* mRNA induced by 1,25D_3_ (Fig 4C). By contrast, co-treatment of 1,25D_3_ and LPS showed a synergy role, and notably increased the expression of *VDR* (Fig 4D).

### Effect of 1,25D_3_ on the expression of AvBDs in PBMCs

**Fig 5:**
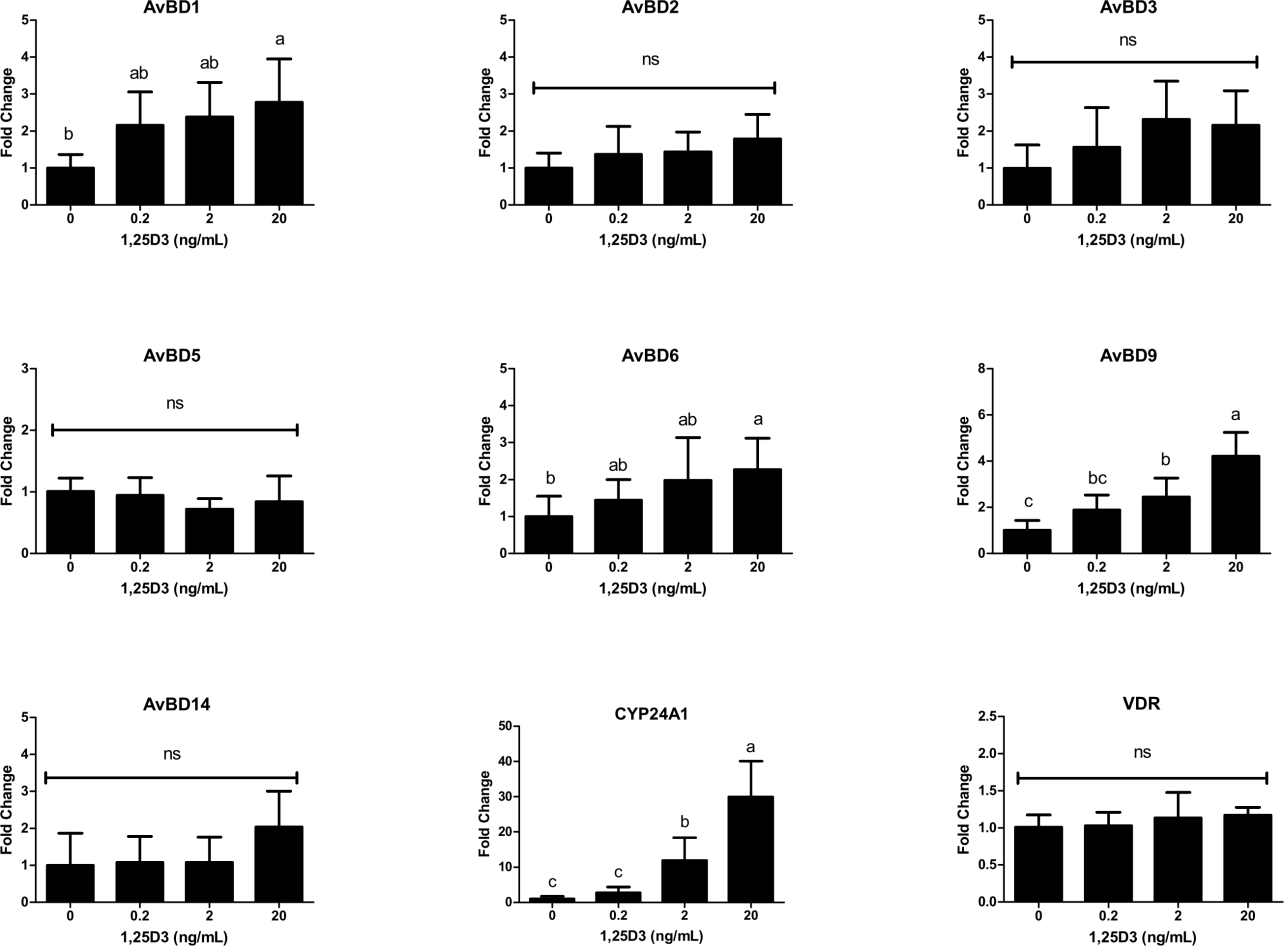
Effects of 1,25D_3_ on the expression of AvBDs in PBMCs. Cells were treated with indicated concentrations of 1,25D_3_ for 24h. The relative gene expression was measured by qPCR and normalized to *GAPDH*. Each bar represents mean ± SD of the results from three independent experiments performed in duplicate. The bars without the same letter indicate differences significant at *P* < 0.05.

In mammals, 1,25D_3_ augmentation of HDPs is widely observed in immunocytes [11, 19, 27]. To investigate the effect of 1,25D_3_ in monocytes, chicken PBMCs were stimulated with different concentration of 1,25D_3_ for 24h. In comparison with CEIEPCs, similar results were also observed in PBMCs (Fig 5). Of the seven expressed AvBD genes [12], 1,25D_3_ significantly increased *AvBD6* and *AvBD9* expression in PBMCs. *AvBD3* was not notably induced in PBMCs at 24h (*P* > 0.05), instead, *AvBD1* was up-regulated about 2.5 fold by 20 ng/mL of 1,25D_3_ (*P* < 0.05). At the same time, 1,25D_3_ increased *CYP24A1* expression in a dose-dependent manner suggesting PBMCs are responding to the stimulation of 1,25D_3_.

### Cycloheximid inhibit 1,25D_3_ mediated expression of AvBDs

**Fig 6:**
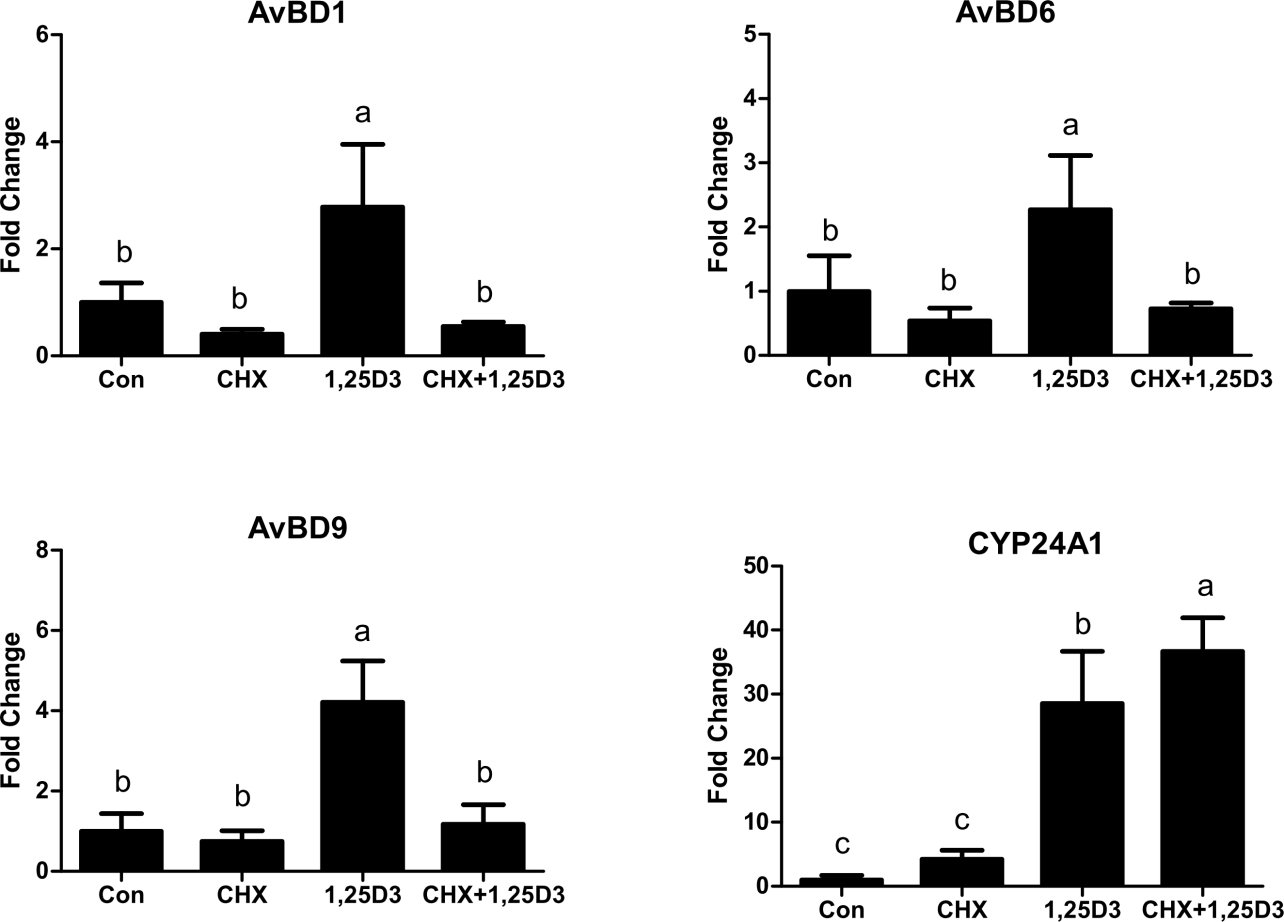
AvBDs are not directly regulated by 1,25D_3_. Chicken PBMCs were pretreated in duplicate with 10 μg/mL of cycloheximide (CHX) for 1h, followed by stimulation with 20 ng/mL 1,25D_3_ for anther 24h. The relative gene expression was measured by qPCR and normalized to *GAPDH*. Each bar represents mean ± SD of the results from three independent experiments performed in duplicate. The bars without the same letter indicate differences significant at *P* < 0.05.

1,25D_3_ exerts its genomic effects through *VDR*. After activation by 1,25D_3_, *VDR* ligand binds the retinoid X receptor (*RXR*) to form VDR:RXR heterodimers which can bind to DNA sequences containing vitamin D response elements (VDREs) and influence the transcription of target genes [28, 29]. In humans, VDREs directly regulate the expression of *cathelicidin* and *human β-defensin* (*HBD) 2*, while an intermediate factor is involved in the regulation of β-defensins in cattle [19]. To reveal whether 1,25D_3_ is able to induce AvBDs expression through VDREs, PBMCs were pre-treated with CHX, the protein translation inhibitor, before 1,25D_3_ treatment. As shown in Fig 6, CHX completed blocked 1,25D_3_-induced up-regulation of *AvBD1*, *AvBD6* and *AvBD9* in PBMCs. In contrast, CHX did not blunt 1,25D_3_-induced expression of *CYP24A1*. Collectively, these results suggest that *VDR* and putative VDREs are not directly involved in the regulation of AvBDs by 1,25D_3_. Instead, those AvBDs appear to be secondary 1,25D_3_ target genes in chicken PBMCs.

## Discussion

In the present study, we have presented the first evidence that 1,25D_3_ is capable of inducing the expression of AvBDs *in vitro*, suggesting that 1,25D_3_-induced synthesis of HDPs not only occurs in mammals, but is also conserved in chickens. This conclusion is further supported by the fact that 1,25D_3_ is able to trigger expression of multiple AvBDs in chicken HD11 macrophages (L. Zhang and G. Zhang unpublished data). This evidence indicates that 1,25D_3_ induction of β-defensins is not specific to mammals. Because all β-defensins emerge from the same ancestor and are conserved across the vertebrate lineage [30, 31], we speculate that 1,25D_3_-mediated β-defensins are widespread in vertebrates. At the same time, specific expression and regulation patterns of AvBDs were also observed. First, not all of the *AvBD* members were detectable in CEIEPCs and PBMCs. Among the 14 AvBDs, three and seven of them were not expressed in CEIEPCs and PBMCs, respectively. Secondly, AvBDs were differently regulated by 1,25D_3_. In CEIEPCs, 1,25D_3_ alone specifically up-regulated *AvBD3*, *AvBD6* and *AvBD9*, while *AvBD4* was induced only in conjunction with LPS. Finally, AvBDs responded to 1,25D_3_ in a cell-specific regulation pattern, as indicated by the inability of 1,25D_3_ to significantly up-regulate *AvBD*3 and *AvBD1* in PBMCs and CEIEPCs, respectively.

In chickens, Vit-D_3_ and its analogues play an immunomodulatory role in the immune system both *in vivo* and *vitro*. In particular, vitamin D-deficiency decreases cutaneous basophil hypersensitivity response to phytohemagglutinin-P [32], while adding extra 25OHD_3_ to diets enhances both the humoral immune response and cell-mediated immune response [33]. Simultaneously, MQ-NCSU cells treated with 1,25D_3_ display increased production of nitric oxide and decreased expression of *IL-1β* as well as chemokine ligand 8 in the presence of LPS [34]. LPS stimulation in HD11 cells concomitantly treated with either 25(OH)D_3_ or 1,25D_3_ have higher amounts of *IL-10* mRNA [35]. Importantly, a recent study found that maternal supplication of 25D_3_ enhanced bactericidal capability in posthatch chickens [36]. However, there is no evidence indicating Vit-D_3_ analogues enhance chicken innate immunity by inducing AvBD expression. Our discovery provides new sight to better understanding the molecular mechanism of Vit-D_3_ and its metabolic productions in chicken immunity.

Due to the presence of potential VDREs in the promoters of chicken AvBDs [23], we hypothesized 1,25D_3_ may increase the relative mRNA abundance of some AvBD genes through VDREs. In this experiment, CHX pretreatment completely inhibited 1,25D_3_-mediated up-regulation of three AvBD genes in PBMCs, however, CHX further increased the expression of *CYP24A1*, the primary VDR/1,25D_3_ target gene [28]. These results indicated the induction of AvBD mRNA is not a direct effect of 1,25D_3_ and other proteins are involved in vitamin D-mediated AvBD response in PBMCs. At present, multiple pathways, proteins and transcription binding motifs have been reported that may contribute to 1,25D_3_-mediated HDP expression. For example, P38, ERK and JNK signaling pathways are involved in *HBD3* expression in keratinocytes [37], while NF-κB signaling pathways are involved in *HBD2* expression in primary monocytes [18]. Meanwhile, 1,25D_3_ treatment increases the levels of Fra1 and c-Fos proteins which enable it to stimulate AP-1 transactivation [37]. In the human THP-1 monocyte cell line, several genes are indirectly induced by 1,25D_3_ through its regulation of several transcription factors [38]. In chicken macrophages, P3, MAPK, JNK and cAMP signaling regulate the expression *AvBD9* [15], suggesting some potential mechanism for further studies. It will be interesting to investigate the mechanisms of 1,25D_3_-induced chicken HDP expression in the future. On the other hand, because LPS induces the section of pro-inflammatory cytokines and is recognized by several chicken *TLR*s [39, 40], the mechanism of 1,25D_3_ in inducing *AvBD4* may be similar to *HBD2* (also known as *DEFB4*) expression in human [18], which means that the activation *TLR*s and cytokines are probably required for the upregulation of *AvBD4* in CEIEPCs. Moreover, because both 1,25D_3_ [41] and LPS [42, 43] are able to alter the histone acetylation which plays a very important role in regulation of HDPs expression [44], we cannot exclude the possibility that histone acetylation is involved in the expression of AvBDs in this study.

The intestinal epithelium is a single-cell layer that generates a physical and biochemical barrier against microorganisms from the external environment. Intestinal epithelial cells secrete various HDPs that not only hinder the growth of microorganisms but also maintain intestinal homeostasis [45–47]. Since intestinal epithelial cells are continually exposed to commensal bacteria and their components, the insensitive of intestinal epithelial cells to LPS is a protective mechanism to avoid eliciting an inflammatory response by the normal external environment [48, 49]. The present results showed that LPS did not significantly trigger the expression of *TLR-4* until it reached a concentration of 800 μg/mL. This suggested that CEIEPCs have developed LPS hyporesponsiveness and tolerance to stimulation by bacterial products. Furthermore, oral administration of HDPs is able to enhance barrier functions and suppress inflammatory diseases [50, 51], indicating that strengthening the expression of AvBDs in intestinal cells is probably beneficial to improve the mucosal antimicrobial barrier and reduce the metabolic consequences of the inflammatory response, especially in newborn chickens.

In this study, although 1,25D_3_ induced the expression of AvBDs, *VDR* was not regulated by different concentrations of 1,25D_3_ in both cell types. Instead, the expression of *VDR* was only induced by 1,25D_3_ in combination with LPS in CEIEPCs. We speculate it is a cell-type specific response and associated with environmental stimuli. In general, increasing the expression of *CYP24A1* could lead to activation of the vitamin D catabolism pathway. Hence, *CYP24A1* serves as a feedback regulator to limit the concentration of 1,25D_3_. In the present study, LPS stimulation inhibited 1,25D_3_ induced up-regulation of *CYP24A1*, meaning it blocked the degradation of 1,25D_3_ and increased the 1,25D_3_ levels compared to cells without LPS stimulation. This may be the related to the effect of 1,25D_3_ on *VDR* and the extra fold change of AvBDs in LPS-activated CIEPCs.

Compared to butyrate that robustly increased HDP expression in chickens, 1,25D_3_ only produced a marginal effect on the expression of AvBDs. It is important to confirm whether 1,25D_3_-induced up-regulation of AvBDs is associated with pathogen clearance and disease resistance in the future. In addition, 1,25D_3_, as well as the vitamin D pathways, are able to interact with other HDP inducers and synergize with specific dietary compounds in HDP induction [13, 52, 53]. For example, in human keratinocytes, butyrate synergizes with 1,25D_3_ to induce the expression cathelicidin and enhance the antimicrobial function [41]. It is thus important to investigate the synergy effects among different HDP inducers in antibiotic alternatives development.

In conclusion, evidence from the present study demonstrates that 1,25D_3_ is able to induce the expression of AvBDs *in vitro* for the first time. Those results indicate that 1,25D_3_ can be used as an antibiotic alternative to enhance chicken immunity in chickens. However, due to the large phylogenetic distance between chicken and mammals, more investigation is needed to detect the mechanisms and cellular signaling pathways of 1,25D_3_ in chickens.
